# Deoxypodophyllotoxin Induces ROS-Mediated Apoptosis by Modulating the PI3K/AKT and p38 MAPK-Dependent Signaling in Oral Squamous Cell Carcinoma

**DOI:** 10.4014/jmb.2207.07012

**Published:** 2022-08-24

**Authors:** Ji-Hye Seo, Goo Yoon, Seryoung Park, Jung-Hyun Shim, Jung-Il Chae, Young-Joo Jeon

**Affiliations:** 1 Department of Dental Pharmacology, School of Dentistry, Jeonbuk National University, Jeonju 54896, Republic of Korea; 2 Department of Pharmacy, College of Pharmacy, Mokpo National University, Muan 58554, Republic of Korea; 3 Disease Target Structure Research Center, Korea Research Institute of Bioscience and Biotechnology (KRIBB), Daejeon 34141, Republic of Korea; 4 Department of Pharmacy, College of Pharmacy and Natural Medicine Research Institute, Mokpo National University, Muan‐Gun, Jeonnam, Republic of Korea

**Keywords:** Deoxypodophyllotoxin, oral squamous cell carcinoma, apoptosis, PI3K/AKT, p38 MAPK

## Abstract

Deoxypodophyllotoxin (DPT), a naturally occurring flavonolignan, possesses several pharmacological properties, including anticancer property. However, the mechanisms underlying DPT mode of action in oral squamous cell carcinoma (OSCC) remain unknown. This study aimed to investigate the anticancer effects of DPT on OSCC and the underlying mechanisms. Results of the MTT assay revealed that DPT significantly reduced the cell viability in a time- and dose-dependent manner. Flow cytometry analysis revealed that DPT induces apoptosis in OSCC cells in a dose-dependent manner. Moreover, DPT enhanced the production of mitochondrial reactive oxygen species (ROS) in OSCC cells. Mechanistically, DPT induced apoptosis in OSCC cells by suppressing the PI3K/AKT signaling pathway while activating the p38 MAPK signaling to regulate the expression of apoptotic proteins. Treatment with SC79, an AKT activator, reversed the effects of DPT on AKT signaling in OSCC cells. Taken together, these results provide the basis for the use of DPT in combination with conventional chemotherapy for the treatment of oral cancer.

## Introduction

In worldwide, oral squamous cell carcinoma (OSCC) is the sixth most common cancer on the basis of approximately 275,000 case results in every year [1]. To cure of this disease, chemotherapy and combination therapy with other drugs are performed principally for the recurrent or metastatic OSCC patients [2]. It has been already well established like this traditional therapy to cure of OSCC patients, but the 5-year survival rate remains less than 50% [3]. Therefore, the development of new effective chemotherapeutic agents for OSCC might help in improving the survival rate of patients with OSCC.

**Fig. 1:**
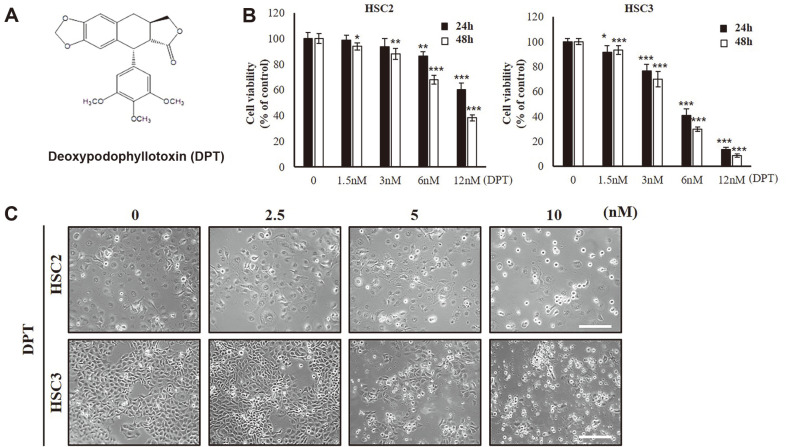
Effect of DPT on cell viability of OSCC cell lines. (**A**) Chemical structure of DPT. (**B**) HSC2 and HSC3 cells were treated with 1.5, 3, 6, and 12 nM DPT for 24 and 48 h. Cell viability was assessed using the MTT assay according to manufacturer’s instructions. Data represent mean percentage levels ± SD, compared to DMSO-treated control cells, paired *t*-test (n = 3) (*p < 0.05, **p < 0.01, ***p < 0.001). (**C**) Morphology of HSC2 and HSC3 cells with or without DPT treatment for 24 h. Scale bar, 100 μm.

Deoxypodophyllotoxin (DPT), the main lignin in *Anthriscus sylvestris* L. (Hoffm) (Fig. 1A), is a natural flavonolignan with antitumor properties. DPT preventions microtubule assembly by directly binding to tubulin [4, 5], promotes cell cycle arrest [6], induces apoptosis [7-9], and stimulates cytoskeleton remodeling by activating the 5'-AMP-activated protein kinase-associated signaling pathways [10]. Studies showed that DPT inhibits the osteosarcoma [11], breast cancer [12], cholangiocarcinoma [13], and glioblastoma [13] cell growth. However, there are no studies on the anticancer effects of DPT in OSCC.

The AKT/p38 MAPK signaling pathway is participate for the many kinds of cancer development, including the breast cancer [14], liver cancer [15], lung cancer [16], colorectal cancer [17], glioma [18], and gastric cancer [19]. MAPKs comprise three major groups (ERKs, p38 MAPKs, and JNKs). These kinases are involved in several kinds of signal by activation of diverse stimuli and provide important roles in multicellular organisms such as apoptosis, cell proliferation, and differentiation [20-22]. Phosphoinositide 3-kinase (PI3K) plays a critical role, which can contribute in cancer cell growth and cellular functions according to extracellular signals [23]. The main function of PI3K is to activate protein kinase B (AKT), which in turn activates downstream proteins involved in promoting cell proliferation through regulating apoptosis and cell cycle [24]. AKT can suppress the p38 MAPK and JNK signaling pathways to inhibit apoptosis [25]. In addition, AKT decreased the activation of apoptosis signaling regulating kinase 1 (ASK1) by phosphorylating Ser-83 residue. Along with inactivation of ASK1, mitogen-activated protein kinase 4 (MKK4), MKK7, MKK3, or MKK8 are unable to activate, which are required for JNK and p38 MAPK signaling pathways activation [26].

Herein, we examined whether DPT could inhibit the growth of OSCC cells. We demonstrated that DPT inhibits cell proliferation by modulating the PI3K/AKT and p38 MAPK signaling pathways, thereby inducing apoptosis. Our results provide insights into the therapeutic efficacy of DPT in OSCC.

## Materials and Methods

### Cell Culture and Reagents

Human oral squamous cancer cell lines, HSC2 and HSC3, were obtained from Hokkaido University (Hokkaido, Japan). HSC2 and HSC3 cells were maintained in Dulbecco’s modified Eagle’s medium (DMEM) (Thermo Scientific, USA) containing 10% heat-inactivated fetal bovine serum, and penicillin-streptomycin (100 U/ml)(Thermo Scientific) at 37°C with 5% CO_2_ in a humidified atmosphere. DPT was purchased from Sigma-Aldrich (USA) and SC79 was purchased from MedChemExpress. 3-(4, 5-dimethylthiazol-2-yl)-2, 5-diphenyl tetrazolium bromide (MTT) was purchased from Duchefa (Netherlands). Antibodies specific to PARP, p-PI3K, p-AKT, AKT, p-p38 MAPK, cleaved caspase-3, Bax, Bak, Bik, and Bim were purchased from Cell Signaling Technology (USA). Antibodies against p21, Mcl-1, GAPDH, and β-actin were obtained from Santa Cruz Biotechnology (USA). Antibodies against PI3K and p38 MAPK were obtained from ABclonal Biotech Co. Ltd. (USA).

### MTT Assay

Cells were plated in 96 well-plates and treated with different concentrations of DPT for 24 and 48 h. After, 20 μl MTT (0.5 mg/ml) was added to each well and incubated for 90 min in a humidified incubator with 5% CO_2_ and 37°C. Absorbance was measured using the Epoch microplate spectrophotometer (BioTek Instruments, Inc., USA) at 540 nm.

### DAPI Staining

Chromatin condensation and fragmentation were analyzed by nucleic acid staining with 4′-6-diamidino-2-phenylindole (DAPI). Briefly, DPT-treated HSC2 and HSC3 cells were harvested by trypsinization and fixed in 100% methanol at room temperature (RT) for 20 min. The cells were spread on slides, stained with the DAPI solution (2 μg/ml), and analyzed using the EVOS FL Auto 2 imaging system (Thermo Scientific).

### Flow Cytometry

Single cell suspension was prepared using trypsin-EDTA (0.25%). For apoptosis assay, cells were suspended in 1× binding buffer (BD Biosciences, USA) and incubated with FITC-conjugated annexin V and 7-AAD (BD Biosciences) for 25 min at RT. Cells were analyzed using BD FACSVerse (BD Biosciences). For the detection of superoxide and reactive oxygen species (ROS), cells were incubated with MitoSOX Red mitochondrial superoxide indicator (Thermo Fisher Scientific) for 10 min at 37°C and analyzed using BD FACSVerse (BD Biosciences). Data were analyzed using the FlowJo software (ver. 10).

### Western Blot Analysis

DPT-treated HSC2 and HSC3 cells were washed twice with ice-cold phosphate buffered saline (PBS) and harvested in ice-cold M-PER mammalian protein extraction reagent (Thermo Scientific) containing cOmplete protease inhibitor cocktail tablets (Roche, Switzerland) and PhosSTOP phosphatase inhibitor cocktail tablets (Roche). Protein concentration was measured using the BCA protein assay kit (Pierce Biotechnology, USA). Protein samples were separated by SDS-PAGE and transferred onto the Immobilon-P PVDF membrane (Millipore, USA) using the Wet/Tank blotting system (Bio-Rad Laboratories, Inc., USA). The membrane was blocked with 5% skimmed milk (Difco Skim Milk, BD Biosciences, USA) at RT for 30 min and then incubated with primary antibodies overnight at 4°C. Membranes were then incubated with horseradish peroxidase (HRP)-conjugated secondary antibodies (Cell Signaling Technology). To detect the HRP signals, ECL Select Western blotting detection reagent (GE Healthcare, USA) was used. Images of the protein bands were acquired using the LAS-3000 imaging system (Fujifilm, Japan).

### Statistical Analysis

All data are presented as mean ± standard error of means (SEM) obtained from three independent experiments. Statistical significance was assessed using the Student’s *t*-test. *p*< 0.05 was considered statistically significant.

## Results

### DPT Inhibits OSCC Cell Growth

The structure of DPT is shown in Fig. 1A. To determine the effect of DPT on OSCC cell proliferation and viability, MTT assay was performed. HSC2 and HSC3 cells were treated with different concentrations of DPT (1.5, 3, 6, and 12 nM) for 24 and 48 h. The IC_50_ value of DPT at 48 h was found to be 10 nM in HSC2 and 4 nM in HSC3 cells. Thus, DPT decreased the proliferation of HSC2 and HSC3 cells in a concentration- and time-dependent manner (Fig. 1B). In addition, morphological changes following DPT treatment were analyzed using an optical microscope. After 24 h of DPT treatment, apoptotic cells with round or irregular shapes and cytoplasmic blebbing were observed (Fig. 1C). These results suggested that DPT inhibits the growth of HSC2 and HSC3 cells.

### DPT Treatment Induces Apoptosis in OSCC Cells

**Fig. 2:**
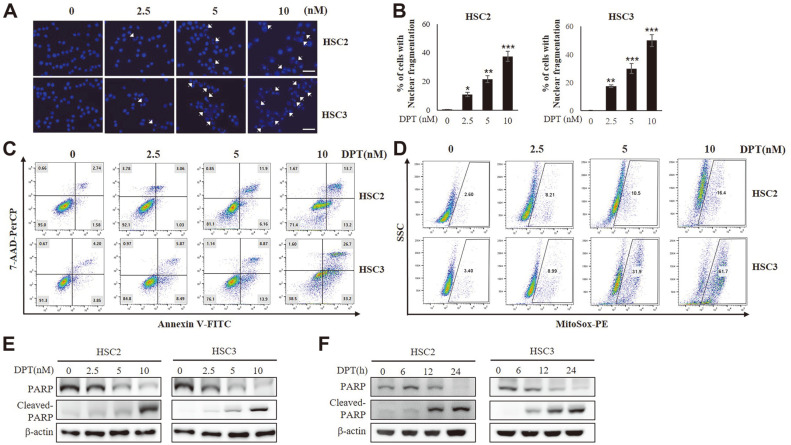
DPT induces apoptosis in OSCC cells. (**A**) HSC2 and HSC3 cells were treated with 2.5, 5, and 10 nM DPT for 24 h and then cells from each well were harvested to examine DNA damage by DAPI-staining, as described in Materials and Methods. Arrows indicate nuclear condensation and DNA fragmentation. Scale bar 100 μm. (**B**) Apoptotic nuclear condensation and DNA fragmentation were quantified. Data represent the mean percentage levels ± SD (n = 3) (*p < 0.05; **p < 0.01; ***p < 0.001). (**C**) DPT-induced apoptotic cell death was analyzed using the Annexin V/7-AAD staining. Cells were treated with 0 (DMSO only), 2.5, 5, and 10 nM DPT for 24 h. Annexin V (+)/7AAD(+) and Annexin V(+)/7AAD (-) were defined as annexin V (+) for apoptosis. (**D**) ROS levels in DPT treated cells were analyzed by MitoSOX-based flow cytometry. Representative flow cytometry-based ROS patterns in DPT treated cells. (**E**) HSC2 and HSC3 cells were treated with 2.5, 5, and 10 nM DPT for 24 h. Whole well extracts were prepared, separated on SDS-PAGE, and subjected to Western blot for PARP. β-actin was used as the loading control. (**F**) Time-dependent effects of DPT on PARP expression in HSC2 and HSC3 cells were evaluated by analyzing the PARP expression at every 6 h interval for 24 h.

To determine whether DPT induces apoptosis in the two OSCC cell lines, we examined the nuclear morphology following DPT treatment. The nuclei were stained using DAPI that enabled us to visualize nuclear condensation and perinuclear apoptotic bodies (Fig. 2A). Results showed that DPT treatment increased the nuclear condensation in HSC2 and HSC3 cells (Fig. 2B). Further, apoptosis assay was performed using annexin V/7-AAD double staining. Results showed that DPT treatment increased the ratio of early to late apoptotic cells in a dose-dependent manner (Fig. 2C). Moreover, total and mitochondrial ROS levels were increased in HSC2 and HSC3 cells after 24 h of DPT treatment (2.5, 5, and 10 nM) (Fig. 2D). Next, we evaluated the levels of cleaved PARP by Western blotting. As shown in Figs. 2E and 2F, PARP cleavage was induced by DPT in a dose- and time-dependent manner. These results indicate that DPT treatment enhances ROS production in HSC2 and HSC3 cells leading to apoptosis.

### DPT Induces Apoptosis by Modulating the PI3K/AKT and p38 MAPK Signaling Pathways in OSCC

**Fig. 3:**
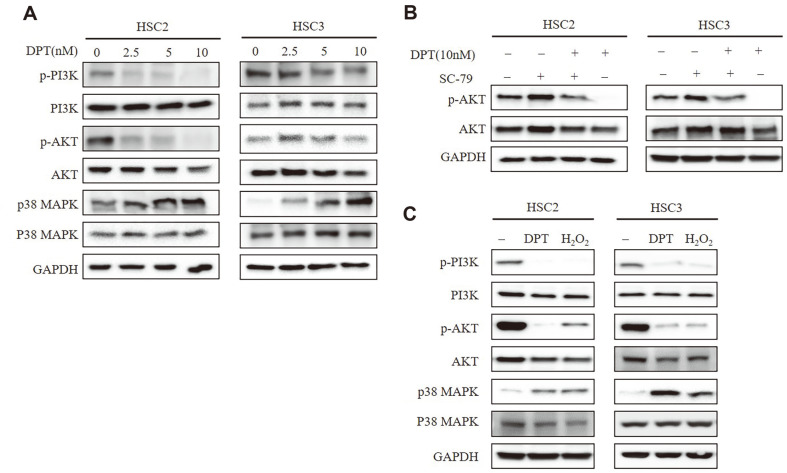
Effects of DPT on PI3K/AKT and p38 MAPK signaling in OSCC cells. (**A**) HSC2 and HSC3 cells were treated with DPT for 24 h. Protein levels of p-PI3K, PI3K, p-AKT, AKT, p-P38 MAPK, and p38 were assessed by Western blotting. (**B**) HSC2 and HSC3 cells were pretreated with or without SC-79 (10 μM) followed by DPT treatment, and p-AKT and AKT levels were determined by Western blotting. GAPDH served as the loading control. (**C**) HSC2 and HSC3 cells were treated with DPT (10 nM) and H_2_O_2_ (400 uM) for 24 h. Protein levels of p-PI3K, PI3K, p-AKT, AKT, p-P38 MAPK, and p38 MAPK were assessed by Western blotting. GAPDH served as the loading control.

The AKT- and MAPK-mediated signaling pathways are participated in suppression of tumor and apoptosis sensitization [26]. Dysregulated activation of these pathways is considered carcinogenic. Therefore, we investigated the effects of DPT on PI3K/ATK and p38 MAPK signaling pathways in OSCC. Results showed that DPT markedly downregulated the p-AKT levels by inhibiting PI3K, suggesting that DPT suppresses this survival pathway (Fig. 3A). Interestingly, a significant increase in the phosphorylated p38 MAPK level was observed in DPT-treated HSC2 and HSC3 cells. To further confirm these findings, cells were treated with the AKT agonist SC79 and the levels of AKT signaling proteins were analyzed. Results demonstrated that p-AKT levels were significantly increased in SC79 and DPT co-treated cells compared to DPT treated cells (Fig. 3B). As shown in Fig. 2D, given that DPT increased mitochondrial ROS, we next evaluated the effect of ROS on PI3K/AKT and p38 MAPK signaling. We induced ROS by treatment with H_2_O_2_ and confirmed that PI3K/AKT and p38 MAPK signaling was regulated in the H_2_O_2_-treated group, similar to that in the DPT-treated group. These results reveal that ROS-mediated apoptosis in HSC2 and HSC3 cells is induced through regulation of the PI3K/AKT and p38 MAPK signaling pathways after DPT treatment.

### DPT Regulates the Expression of Apoptosis-Related Proteins in OSCC

**Fig. 4:**
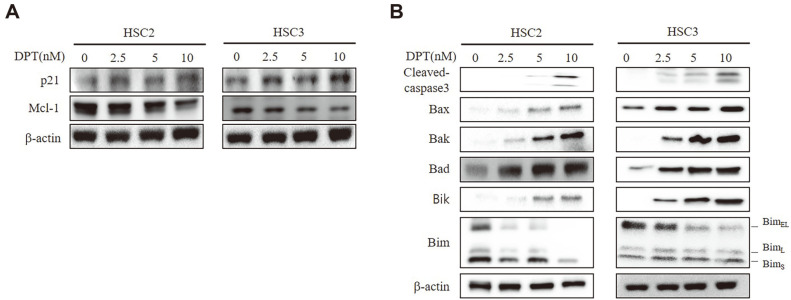
Effect of DPT on apoptosis in OSCC cells. (**A**) HSC2 and HSC3 cells were treated with DPT (2.5, 5, and 10 nM) for 24 h. Western blot analysis was performed using antibodies against p21 and Mcl-1. β-actin served as the loading control. (**B**) HSC2 and HSC3 cells were treated with DPT (2.5, 5, and 10 nM) for 24 h. Western blot analysis was performed using antibodies against cleaved caspase 3, Bax, Bak, Bad, Bik, and Bim. β-actin served as the loading control.

**Fig. 5:**
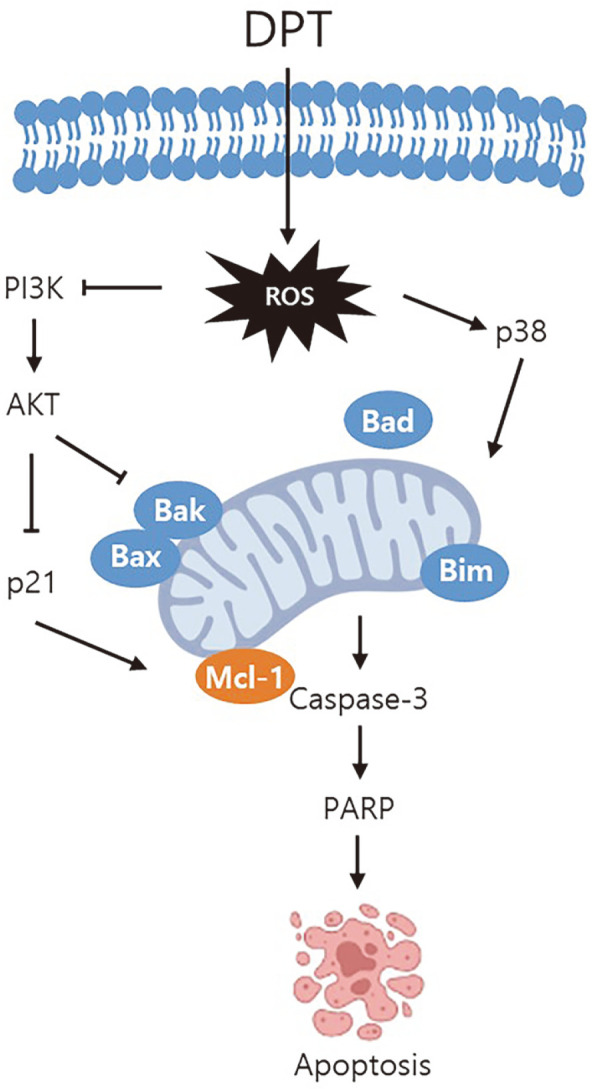
DPT induced apoptosis by suppressing PI3K/AKT and activating p38 MAPK in OSCC cells.

To validate the role of DPT in cellular function regulation, we focused on the downstream targets of PI3K/AKT and p38 MAPK signaling pathways and apoptosis-related proteins. Results showed that the expression of the cell cycle arrest protein p21 was markedly enhanced in a dose-dependent manner following DPT treatment, whereas the expression of the cell survival- and proliferation-related protein Mcl-1 was decreased (Fig. 4A). In addition, DPT-treatment dose-dependently increased the activation of caspase-3 and regulation the expression of Bax, Bak, Bad, Bik, and Bim in HSC2 and HSC3 cells (Fig. 4B). In conclusion, these results show that DPT inhibits OSCC progression by regulating the PI3K/AKT and p38 MAPK signaling pathways. In Fig. 5, the potential mechanisms of DPT are summarized.

## Discussion

This study was designed to validate the anticancer effects of DPT in OSCC and to elucidate the underlying molecular mechanisms. The main findings of this study are as follows: i) DPT reduces cell viability and induces apoptosis in OSCC cells; and ii) DPT modulates the PI3K/AKT and p38 MAPK signaling pathways to exert antitumor effects.

OSCC accounts for over 90% of oral cancers [27]. Despite the development of multiple clinical approaches, including chemotherapy, radiation therapy, surgical resection, or their combination, OSCC remains the most aggressive type of malignancy and patients with OSCC have a low survival rate. Therefore, development of novel therapeutic approaches and more effective medicine for the cure of OSCC is exigently required. Although the anticancer properties of DPT are previously studied, the underlying mechanisms remain poorly understood. In the present study, DPT inhibited cell viability in a concentration- and time-dependent manner. Moreover, apoptosis was promoted dependent with DPT concentration by inducing apoptotic bodies and DNA fragmentation. Natural products exhibit their anticancer activities by inducing apoptosis [28]. Upon apoptosis initiation, the intracellular ROS levels are increased [29]. In the case of the ROS production rate is much faster than its clearance rate, cellular apoptosis is prompted [30]. In our result, we could be observed that increase of mitochondrial ROS levels in OSCC cells via DPT treatment.

The development of OSCC is often related to change in molecular level of the receptor tyrosine kinase (RTK), PI3K/AKT, and p53 pathway, and G1/S cell cycle transition [31-33]. The PI3K family kinases are lipid kinases comprising of the catalytic subunits (p110α, p110β, p110δ and regulatory subunits (p85α, p85β, p55γ, p55α, p50α) and are downstream of RTKs. Activated PI3K can convert phosphatidylinositol 3,4-bisphosphate (PIP2) into 3,4,5-triphosphate (PIP3); PIP3 binds to phosphoinositide-dependent kinase-1 (PDK1) and serves as a second messenger capable of phosphorylating AKT at Thr308.

Following RTKs activation, AKT regulates multiple cellular processes, including proliferation, metabolism, invasion, and apoptosis [34]. The AKT protein contains two phosphorylation sites, Ser473 and Thr308, which must be phosphorylated by phosphoinositide-dependent kinase 1 and the rapamycin-insensitive complex, respectively, to induce AKT activation. AKT phosphorylation is widely regarded as a marker of PI3K activity [35]. The MAPK signaling controls basic cellular processes in cancer, such as apoptosis, proliferation, and response to chemotherapeutic drugs [36, 37]. Presently, mammalian MAPKs are divided into three major groups: ERKs (p44/p42), JNKs, and p38. JNK and p38 are participated in regulating apoptosis, whereas ERK is involved in regulating cell proliferation, migration, and senescence [38, 39]. Our novel findings indicated that DPT inhibits PI3K/AKT activity and activates the p38 signaling pathway in OSCC. Further, SC79, an AKT-specific agonist was used to validate our findings. Results showed that SC79 abrogates the inhibitory effects of DPT on OSCC, confirming that DPT modulates the PI3K/AKT signaling pathway.

Furthermore, we analyzed the effects of DPT on p21, which is closely related with cell cycle arrest and cell survival. p21 is a well characterized negative regulator of cell cycle progression, and its interaction with the cyclin-dependent kinases (CDKs) is responsible for G1 phase arrest [40]. Therefore, we hypothesized that therapeutic agents that positively regulate CDKs, might also suppress malignancy by inhibiting the cell cycle progression. Previous studies showed that the anti-apoptotic Bcl-2 family protein Mcl-1 is associated with cancer progression and metastasis [41, 42]. Downregulation of Mcl-1 promotes apoptosis in various cancer cells [43, 44]. In this study, we discover that the p21 was significantly increased while that of Mcl-1 was markedly decreased by DPT in a concentration-dependent manner. Consistently, DPT treatment reduced Bim expression and increased the expression of Bax, Bak, Bad, and Bik. DPT also induced PARP and caspase-3 activation, suggesting that DPT causes apoptosis in OSCC cells via intrinsic cell death pathways.

In conclusion, DPT exhibits potent anti-cancer effects in OSCC and promotes apoptosis by suppressing the PI3K/AKT signaling and activating the p38 MAPK signaling pathway. Thus, DPT could be a potential therapeutic agent that can be further developed for clinical trials in patients with OSCC.
