# Corrigendum to: Deoxypodophyllotoxin Induces ROSMediated Apoptosis by Modulating the PI3K/AKT and p38 MAPK-Dependent Signaling in Oral Squamous Cell Carcinoma

**DOI:** 10.4014/jmb.2022.3210.1355

**Published:** 2022-10-28

**Authors:** Ji-Hye Seo, Goo Yoon, Seryoung Park, Jung-Hyun Shim, Jung-Il Chae, Young-Joo Jeon

**Affiliations:** 1 Department of Dental Pharmacology, School of Dentistry, Jeonbuk National University, Jeonju 54896, Republic of Korea; 2 Department of Pharmacy, College of Pharmacy, Mokpo National University, Muan 58554, Republic of Korea; 3 Disease Target Structure Research Center, Korea Research Institute of Bioscience and Biotechnology (KRIBB), Daejeon 34141, Republic of Korea; 4 Department of Pharmacy, College of Pharmacy and Natural Medicine Research Institute, Mokpo National University, Muan‐Gun, Jeonnam, Republic of Korea

In the article titled “Deoxypodophyllotoxin Induces ROS-Mediated Apoptosis by Modulating the PI3K/AKT and p38 MAPK-Dependent Signaling in Oral Squamous Cell Carcinoma”, the authors noticed that the protein names was given incorrectly in the Figure 3A and C. The protein name p38 MAPK should be changed to p-p38 MAPK in Figures A and C in the paper.

The correct ‘Figure 3A and 3C’ are now available online.

